# The COM-Poisson Process for Stochastic Modeling of Osmotic Inactivation Dynamics of *Listeria monocytogenes*

**DOI:** 10.3389/fmicb.2021.681468

**Published:** 2021-07-09

**Authors:** Pierluigi Polese, Manuela Del Torre, Mara Lucia Stecchini

**Affiliations:** ^1^Polytechnic Department of Engineering and Architecture, University of Udine, Udine, Italy; ^2^Department of Agricultural, Food, Environmental and Animal Sciences, University of Udine, Udine, Italy

**Keywords:** *Listeria monocytogenes*, osmotic inactivation, modeling, variation, Poisson, Conway-Maxwell-Poisson, population levels, Monte Carlo

## Abstract

Controlling harmful microorganisms, such as *Listeria monocytogenes*, can require reliable inactivation steps, including those providing conditions (e.g., using high salt content) in which the pathogen could be progressively inactivated. Exposure to osmotic stress could result, however, in variation in the number of survivors, which needs to be carefully considered through appropriate dispersion measures for its impact on intervention practices. Variation in the experimental observations is due to uncertainty and biological variability in the microbial response. The Poisson distribution is suitable for modeling the variation of equi-dispersed count data when the naturally occurring randomness in bacterial numbers it is assumed. However, violation of equi-dispersion is quite often evident, leading to over-dispersion, i.e., non-randomness. This article proposes a statistical modeling approach for describing variation in osmotic inactivation of *L. monocytogenes* Scott A at different initial cell levels. The change of survivors over inactivation time was described as an exponential function in both the Poisson and in the Conway-Maxwell Poisson (COM-Poisson) processes, with the latter dealing with over-dispersion through a dispersion parameter. This parameter was modeled to describe the occurrence of non-randomness in the population distribution, even the one emerging with the osmotic treatment. The results revealed that the contribution of randomness to the total variance was dominant only on the lower-count survivors, while at higher counts the non-randomness contribution to the variance was shown to increase the total variance above the Poisson distribution. When the inactivation model was compared with random numbers generated in computer simulation, a good concordance between the experimental and the modeled data was obtained in the COM-Poisson process.

## Introduction

Providing an easy way to access prediction, the deterministic approach to the description of microbial populations has long been successful in managing food safety. Process and formulation of foods have long benefited from non-thermal inactivation (NTI) models, which represent valuable tools for controlling pathogens (Lindqvist and Lindblad, 2009). However, the point estimates provided by the deterministic models, which do not take into account variability and uncertainty, may be insufficient for a more realistic estimation of microbial behavior (Membré et al., 2006; Koutsoumanis and Angelidis, 2007; Couvert et al., 2010; Augustin et al., 2011). The European Food Safety Authority defines uncertainty as variation associated with the lack of knowledge or the use of imprecise data, and variability as variation in the response of the individual cells within the population, which cannot be reduced based on knowledge [European Food Safety Authority (EFSA) Scientific Committee, Benford et al., 2018; Schendel et al., 2018]. Numerical estimation of microorganisms can be affected by different sources of uncertainty introduced by the experimental procedures, which can include serial dilution and viable cell enumeration (Garre et al., 2019). Likewise, there are numerous sources of variability, which are associated to both the microorganism and the environment, affecting microbial behavior (Koutsoumanis et al., 2016; Koyama et al., 2016; Koutsoumanis and Aspridou, 2017; Aspridou et al., 2019).

The importance of the separation of variability and uncertainty has long been recognized for quantitative microbial risk assessment (QMRA) purposes (Nauta, 2000; den Besten et al., 2018), but the combined description of variability and uncertainty can also play an important role in the valid prediction of bacterial behavior, which is crucial for management interventions (Aguirre et al., 2009; Membré and van Zuijlen, 2011; Aspridou and Koutsoumanis, 2015; Zwietering, 2015; Koyama et al., 2019).

Poisson distribution or the Poisson stochastic process, which refers to the Poisson distribution, have been applied to describe variation in the number of survivors in thermal and non-thermal processes (Aguirre et al., 2009; Koyama et al., 2017). A major assumption of the Poisson model is that the variance is equal to the mean. This assumption is violated if the variance is greater or lower than the mean and, therefore, there is evidence of over or under-dispersion, respectively (Jarvis, 2016).

Over-dispersion is defined as the extra variation occurring in count data modeling which is not explained by the Poisson distribution alone (Rigby et al., 2008). As summarized by Payne et al. (2017) over-dispersion is a common feature in real count data and may occur due to population heterogeneity, correlation, omission of important covariates in the model, the presence of outliers, zero inflation, or other reasons. Under-dispersion may also be encountered in real applications and can be caused by model-overfitting or seen in datasets with small sample values (Sellers and Morris, 2017). Under-dispersion can also be associated with the presence of zero counts in a data set (Sellers and Raim, 2016; Tin, 2008).

Naturally occurring bacteria often exist as social communities and live in spatially structured habitats where spatial heterogeneity is generated (Eriksen et al., 2020). The under-dispersion pattern is characterized by a regular spatial distribution, indicating repulsion, while over-dispersion reflects clustering, indicating aggregation, and both can result from microbial growth or inactivation (Lynch et al., 2014; Jongenburger et al., 2012b; Jarvis, 2016).

Over and under-dispersion, unless properly handled, can lead to biased inferences (Payne et al., 2017; Sellers and Morris, 2017).

The context of this study is a planktonic culture and not a solid or a gelled system where the immobilization of bacteria has an effect on their distribution (Baka et al., 2016, 2017; Jeanson et al., 2011, 2015). However, even the planktonic-cell spatial distribution can show heterogeneity, as happens in the case of aggregate formation. In fact, the assumption that planktonic bacterial cells are by definition not aggregated has been challenged and evidence has been provided of mechanical connections between bacterial cells in diluted planktonic suspensions (Sretenovic et al., 2017). Thus, when randomly distributed cells form aggregates a clustered distribution (over-dispersion) can be generated (Gao et al., 2016).

In this study, the random component of the total variation, which can be represented by the Poisson distribution was referred to as randomness (Lynch et al., 2014; Koyama et al., 2017), whereas the unexplained part of variation (over or under-dispersion), which represents the departure from randomness was referred to as non-randomness (Hui et al., 2010).

When the dispersion pattern of cells deviated from the homogeneous Poisson process other models could be applied to capture count dispersion. For example, the Poisson-Lognormal distribution was found appropriate for the representation of high microbial counts, while the Poisson-gamma (or negative binomial) distribution was better for the characterisation of low microbial counts and for highly clustered microbial data, but both can cope only with over-dispersion (Gonzales-Barron et al., 2010, 2014; Gonzales-Barron and Butler, 2011; Jongenburger et al., 2012b).

When dealing with pathogenic bacteria, variability in concentration of the raw material can be high (European Food Safety Authority (EFSA), 2012). By applying an inactivation process it is likely to result in low and zero counts (European Food Safety Authority (EFSA), 2018). It follows that distributions flexible enough to deal with low, medium or higher counts and suitable for over and under-dispersed data could be more appropriate. The Conway–Maxwell-Poisson (COM-Poisson) (Conway and Maxwell, 1962) distribution, which is a two-parameter generalization of the Poisson distribution, proved to be a useful and elegant model for fitting count data ranging from high to low, including zero, with an unlimited range of dispersion (Sellers and Shmueli, 2010; Francis et al., 2012; Gupta et al., 2014). A more detailed overview of the history, features and applications of COM-Poisson distribution is in Shmueli et al. (2005).

Therefore, in consideration of the variation of the numbers of pathogens usually occurring in food during processing, it would be of importance to derive a statistical model from different population size experiments, to capture the variation in the response of a pathogen, such as *Listeria monocytogenes*, in an osmotic inactivation process, such as that used for processed meat products. For these products, the micro-organisms of concern are both *Salmonella* spp. and *L. monocytogenes*. However, *L. monocytogenes* proved to be less susceptible to manufacturing processes than *Salmonella*, with water activity (a_w_) being a key factor in the survival of the pathogen (Mataragas et al., 2015; Nightingale et al., 2006). Indeed, the severity of *Listeria* illness and the possibility of infection from low doses underlines the necessity to control not only its frequency but also its different levels of contamination (Buchanan et al., 2017; Polese et al., 2017; Radoshevich and Cossart, 2018).

The main objective of the present study was to propose a regression model able to deal with a wide range of dispersion levels, in order to describe the variation in osmotic inactivation of *L. monocytogenes* Scott A at different initial cell levels. To achieve this goal, we investigated whether the variation in survival of *Listeria* numbers followed the theoretical COM-Poisson distribution, which extends the Poisson distribution by adding a parameter to model over- and under-dispersion. This fitting procedure provided a framework able to incorporate the randomness and non-randomness contributors to variation allowing for a more accurate quantification of the survivor dispersion.

## Materials and Methods

### Preparation of *L. monocytogenes* Cells

*Listeria monocytogenes* strain Scott A, serotype 4b, a virulent clinical isolate from a food-borne listeriosis outbreak in 1983 (Briers et al., 2011) was used in this study. This strain was selected for its tolerance to stress conditions encountered in food, which included exposure to conditions of high osmolarity (Becker et al., 1998; Bucur et al., 2018; Durack et al., 2013). The *L. monocytogenes* strain was taken from porous cryobeads (Microbank^TM^, Pro-Lab Diagnostic, Richmond Hill, ON, Canada) that had been stored at −30°C. The strain was cultured in 10 mL Brain Heart Infusion (BHI, Oxoid Ltd., Hampshire, United Kingdom) broth incubated overnight at 30°C. No osmotic adaptation was adopted that could have increased phenotypic heterogeneity (Kapetanakou et al., 2019). This first inoculum of approximately 4.3 × 10^9^ CFU/mL was thoroughly vortexed (Vortex mixer, Velp Scientifica, Usmate, Italy) and then diluted in saline/peptone water [8.5 g L^–1^ NaCl (J.T. Baker^TM^, Baker analyzed^®^ A.C.S, Thermo Fisher Scientific, Waltham, MA, United States) and 1 g L^–1^ Bacteriological Peptone (Oxoid Ltd)] to attain different sub-inoculum levels of approximately 2.2 × 10^5^ CFU/mL, 2.2 × 10^4^ CFU/mL and 2.2 × 10^3^ CFU/mL. Aliquots of 0.07 or 0.14 mL of the latter inoculum solutions were further diluted in the osmotic challenge medium (see section “Osmotic Inactivation Trials”) to obtain final *Listeria* concentrations of approximately 2 × 10 CFU/mL (low inoculum: L), 10^2^ CFU/mL (medium inoculum: M) and 10^3^ CFU/mL (high inoculum: H).

### Osmotic Inactivation Trials

The osmotic challenge medium used was BHI broth supplemented with 134 g L^–1^ NaCl to produce a reduction of the a_w_ that mimicked a representative condition of processed meat products subjected to salt treatment. The a_*w*_ was assessed using an Aqua Lab CX2 instrument (Decagon Devices, Inc., Pullman, Washington, United States) and the measured a_*w*_ value was 0.913 ± 0.001. The pH was adjusted to 6.6 with HCl 1M (Carlo Erba Reagents, Val-de-Reuil, France) using an HI1131B (Hanna instruments, Verona, Italy) electrode and an HI5221 pH-meter (Hanna Instruments, Verona, Italy), equipped with a temperature probe. BHI broth supplemented with NaCl was sterilized by filtration (Nalgene Rapid-Flow 0.2 μm aPES membrane, Thermo Fisher Scientific, Waltham, MA, United States), poured in 15 mL aliquots in sterile tubes and stored at 4°C until use. Prior to inoculation, the osmotic challenge medium was pre-warmed at 30°C, with this temperature being maintained throughout the trials. Samples from bacterial suspensions were thoroughly vortexed (Vortex mixer, Velp Scientifica, Usmate, Italy) before taking samples at time intervals (0, 3, 6, 10, 13, and 18 days). The cell density in each solution was determined by surface plating 0.2 mL solution onto BHI (BHI, Oxoid Ltd., Hampshire, United Kingdom) agar plates (five plates for each sample). The colonies were counted after 48 h incubation at 30°C. The enumeration process was conducted omitting serial dilutions for reducing the impact of uncertainty (Garre et al., 2019). The data were expressed as the mean value (CFU in 0.2 mL or Ln CFU in 0.2 mL) for the five replicates of plate counts. Three independent trials were conducted for each *Listeria* concentration tested.

### Autoaggregation Assay

The ability of *L. monocytogenes* to autoaggregate was measured in phosphate buffered saline (PBS) after 20 h culture in BHI, according to the method described by Collado et al. (2008), with some modifications. *Listeria* cultures were harvested by centrifugation, washed twice in PBS, pH 7.1 (10 mM Na_2_HPO_4_, 1 mM KH_2_PO_4_, 140 mM NaCl, 3 mM KCl) and suspended in the same buffer or in the same buffer supplemented with further NaCl to produce a_*w*_ of 0.913 ± 0.001. The optical density (OD_600_ nm) of the homogenized bacterial suspensions were adjusted to 0.3 ± 0.05 with the same buffers listed above. In addition, the dependence on the concentration was tested adjusting the initial OD of the osmotic medium from 0.2 ± 0.05 up to 0.6 ± 0.05. To determine percentage autoaggregation, suspensions were incubated in aliquots at 30°C without vortexing and monitored (OD_600_ nm) at 24 and 72 h. Autoaggregation was assessed by a decrease in the OD_600_ indicating an increase in bacterial sediments that settle at the bottom of culture tubes. The aggregation percentage was expressed as [(1 – (OD_*Time*_/OD_0_)) × 100] where OD_*Time*_ represents the optical density of the mixture at the different incubation times, i.e., at 24 h and at 72 h, while OD_0_ is the optical density at time 0 h.

### The NTI Models

The change of survivors over inactivation time was described as an exponential function (1):

(1) $$\begin{array}{c} \mu_{\text{t}}=\exp\left(\text{a}+\text{b}\cdot t\right) \end{array}$$

where **μ_*t*_** is the centering parameter of the Poisson (i.e., the mean number of survivors at time t) or the COM-Poisson distribution (Shmueli et al., 2005; Guikema and Goffelt, 2008; Sellers and Shmueli, 2010; Supplementary Box 1); **a** is a regression parameter, which corresponds to LnN_0_; **b** represents the inactivation rate (day^–1^); **t** (day) is the time of the osmotic treatment.

Equations (2, 3) were used for the Poisson and COM-Poisson processes, respectively:

(2) $$\begin{array}{c} {\text{N}}_{\text{t}}∼POISS\left(\mu_{\text{t}}\right) \end{array}$$

(3) $$\begin{array}{c} {\text{N}}_{\text{t}}∼CMP\left(\mu_{\text{t}},v\right) \end{array}$$

where **N_*t*_** (the observed value) is the realization of **μ_*t*_**; **_∼_** is the tilde symbol which means: has the distribution of; **ν** denotes the COM–Poisson dispersion parameter.

In the COM-Poisson distribution model the mean ***E***[**N_*t*_]** and the variance ***VAR***[**N_*t*_**] of **N_*t*_** can be approximated by eqs. (4a,b) as follows:

(4a,b) $$\begin{array}{c} E\left[{\text{N}}_{\text{t}}\right]\approx \mu_{\text{t}}+\frac{1}{2v}-\frac{1}{2},VAR\left[{\text{N}}_{\text{t}}\right]\approx \frac{1}{v}\mu_{\text{t}} \end{array}$$

where ***E***[**N_*t*_]** is the mean of survivors at time t; **μ_*t*_** is the centering parameter of the COM-Poisson distribution (Supplementary Box 1); ***VAR***[**N_*t*_**] is the variance of **N_*t*_**; ***ν*** is the COM–Poisson dispersion parameter.

Equation (4b) becomes eq. (5):

(5) $$\begin{array}{c} \mu_{\text{t}}\approx vVAR\left[{\text{N}}_{\text{t}}\right] \end{array}$$

Thus, eq. (4a) can be rewritten as (6):

(6) $$\begin{array}{c} E\left[{\text{N}}_{\text{t}}\right]\approx vVAR\left[{\text{N}}_{\text{t}}\right]+\frac{1}{2v}-\frac{1}{2} \end{array}$$

To produce a framework for the variance of **N_*t*_**, it is assumed, as in the negative binomial distribution, that the total population variance is represented by the combination of the randomness and the non-randomness components. The first component corresponds to complete homogeneity and can be represented by the mean, whereas the second component represents the non-random variation and is given by a quadratic function of the mean (El-Shaarawi et al., 1981). Therefore, let the randomness component having a Poisson distribution be equal to the mean***E***[**N_*t*_]** and assume the non-randomness component (corresponding to **c_0_*E***[**N_*t*_]**^2^) to be a quadratic function of the mean (El-Shaarawi et al., 1981; Gonzales-Barron et al., 2010; Gonzales-Barron and Butler, 2011), it follows that (Eq. 7):

(7) $$\begin{array}{c} VAR\left[{\text{N}}_{\text{t}}\right]=E\left[{\text{N}}_{\text{t}}\right]+{\text{c}}_{0}E{\left[{\text{N}}_{\text{t}}\right]}^{2} \end{array}$$

where ***VAR*[N_*t*_]** is the variance of **N_*t*_**; ***E***[**N_*t*_]** is the mean number of survivors at time **t**; **c_0_** is the non-randomness variance parameter.

Equation (6) becomes (8) as follows:

(8) $$\begin{array}{c} E\left[{\text{N}}_{\text{t}}\right]\approx v\left(E\left[{\text{N}}_{\text{t}}\right]+{\text{c}}_{0}E{\left[{\text{N}}_{\text{t}}\right]}^{2}\right)+\frac{1}{2v}-\frac{1}{2} \end{array}$$

For low values of ***E***[**N_*t*_]**, **ν** is close to 1, giving the Poisson; while for high values of ***E***[**N_*t*_]** (Supplementary Box 2) Eq. (8) is reduced to Eq. (9):

(9) $$\begin{array}{c} E\left[{\text{N}}_{\text{t}}\right]\approx v\left(E\left[{\text{N}}_{\text{t}}\right]+{\text{c}}_{0}E{\left[{\text{N}}_{\text{t}}\right]}^{2}\right) \end{array}$$

Thus, the COM-Poisson dispersion parameter **ν,** was expressed by Eq. (10) which describes the effect of ***E***[**N_*t*_]** on this parameter:

(10) $$\begin{array}{c} v\approx \frac{E\left[{\text{N}}_{\text{t}}\right]}{\left(E\left[{\text{N}}_{\text{t}}\right]+{\text{c}}_{0}E{\left[{\text{N}}_{\text{t}}\right]}^{2}\right)} \end{array}$$

The COM-Poisson dispersion parameter **ν** represents, therefore, the inverse of the variance over mean ratio. From eq. (10), as **N_*t*_** → ∞, **ν** converges to 0.

For a population subjected to osmotic stress, which can facilitate aggregation (Jensen et al., 2007; Schmid et al., 2009; Eshwar et al., 2017) and hence the production of clustered count data that result in over-dispersion (Jongenburger et al., 2012b), an additional non-randomness term occurring over time in response to the osmotic treatment contributed to the total variance (see section “Results”). Since no extensive literature exists on the non-randomness variance, this additional contribution, which was obtained balancing the number of survivors with the initial number of cells, can be regarded as an empirical term. To describe this additional contribution, a number of empirical equations were developed (Supplementary Box 3) and the Akaike information criterion (AIC) (Vrieze, 2012) was used to select the best-fit model under parsimony, which resulted in (11):

(11) $$\begin{array}{c} \varepsilon_{\text{t}}\text{t}E{\left[{\text{N}}_{\text{t}}\right]}^{2}/E{\left[{\text{N}}_{0}\right]}^{\frac{1}{2}} \end{array}$$

where **ε_*t*_** is the additional non-randomness variance parameter;***E***[**N_*t*_]** is the mean number of survivors at time t.

Including the non-randomness contribution, we can write (12):

(12) $$\begin{array}{c} VAR\left[{\text{N}}_{\text{t}}\right]\approx E\left[{\text{N}}_{\text{t}}\right]+{\text{c}}_{0}E{\left[{\text{N}}_{\text{t}}\right]}^{2}+\varepsilon_{\text{t}}\text{t}E{\left[{\text{N}}_{\text{t}}\right]}^{2}/E{\left[{\text{N}}_{0}\right]}^{\frac{1}{2}} \end{array}$$

where ***VAR*[N_*t*_]** is the variance of **N_*t*_**; **c_0_*E***[**N_*t*_]**^2^ is the non-randomness contribution to the variance; **ε_*t*_t *E*[N_*t*_]^2^/*E*[N_0_]^1/2^** is the additional non-randomness contribution to the variance occurring over the treatment time, and **ε_*t*_** is the additional non-randomness variance parameter.

For a population subjected to osmotic inactivation, **ν** can therefore be expressed as (13):

(13) $$\begin{array}{c} v\approx \frac{E\left[{\text{N}}_{\text{t}}\right]}{\left(E\left[{\text{N}}_{\text{t}}\right]+{\text{c}}_{0}E{\left[{\text{N}}_{\text{t}}\right]}^{2}+\varepsilon_{\text{t}}\text{t}E{\left[{\text{N}}_{\text{t}}\right]}^{2}/E{\left[{\text{N}}_{0}\right]}^{\frac{1}{2}}\right)} \end{array}$$

where ***E***[**N_*t*_], c_0_*E***[**N_*t*_]**^2^ and **ε_*t*_t *E*[N_*t*_]^2^/*E*[N_0_]^1/2^** are as above.

The final NTI model in the Poisson process was obtained using Eqs. (1, 2), whereas the model in the COM-Poisson process was obtained using Eqs (1, 3, and 13).

For providing an estimation of **c_0_** and **ε_*t*_** within the COM-Poisson process, a maximum- likelihood regression on all the survival data was conducted (for details, see section “Stochastic Processes and Statistical Tests”). For practical reasons (too time-consuming calculations), the estimation was limited to the shape function (**ν**), holding the centering function (**μ**) fixed to the previously estimated regression values (LnN_0_ and *b* values of each trial).

### Stochastic Processes and Statistical Tests

The Poisson and the COM-Poisson (Shmueli et al., 2005; Guikema and Goffelt, 2008; Sellers and Shmueli, 2010) distributions (Supplementary Box 1) were fitted to the *Listeria* initial counts (178 data, Supplementary Table 1) and to the survival data over time (993 data, Supplementary Table 1), and the parameters of the cell counts distributions were estimated using the maximum-likelihood method (Nelder and Wedderburn, 1972).

The maximum-likelihood coefficient estimates of the Poisson and the COM-Poisson frameworks were obtained from regression by maximizing equation (S6) (Supplementary Box 1) under the constraint **ν** ≥ 0, using Excel Solver add-in (Microsoft Office Excel 2007, v12.0.6611.1000) as a nonlinear optimization tool. The COM-Poisson equations were coded adapting the algorithm proposed by Huntley (2005), in Visual Basic for Application (VBA) and used in Excel workbooks (Supplementary Table 2). Regression parameters, standard errors and related goodness of fit tests were obtained through SolverStat add-in (Comuzzi et al., 2003) by using Fisher Information Matrix and bootstrap (Sellers and Shmueli, 2010). All other used statistical analyses [Log-likelihood (LL) value, likelihood ratio (LR) test (Santner and Duffy, 1989), AIC (Vrieze, 2012), variance-to-mean ratio, ANOVA, mean, variances, standard deviations] were conducted in Excel. The LR test was computed by taking twice the difference in negative log-likelihoods between the full model (COM-Poisson) and the reduced model (Poisson). The parsimony principle, which is not considered by the LR and LL tests, is included by using the AIC method.

Analysis of deviance for generalized linear regression models (Hardin and Hilbe, 2018) was carried out by using Excel from the deviance values obtained with SolverStat add-in (Comuzzi et al., 2003).

Comparison between the observed variances in the number of surviving *Listeria* cells and the predicted variances in the Poisson and the COM-Poisson distributions was also carried out using Excel. The standard error of the experimental variance was calculated as sqrt[2/(n–1)] VAR[N_*t*_] (Harding et al., 2014). The variance estimated under the Poisson assumption was the mean, while the COM-Poisson variance was the mean divided by ν.

### Simulation via Random Number Generation

**FIGURE 1:**
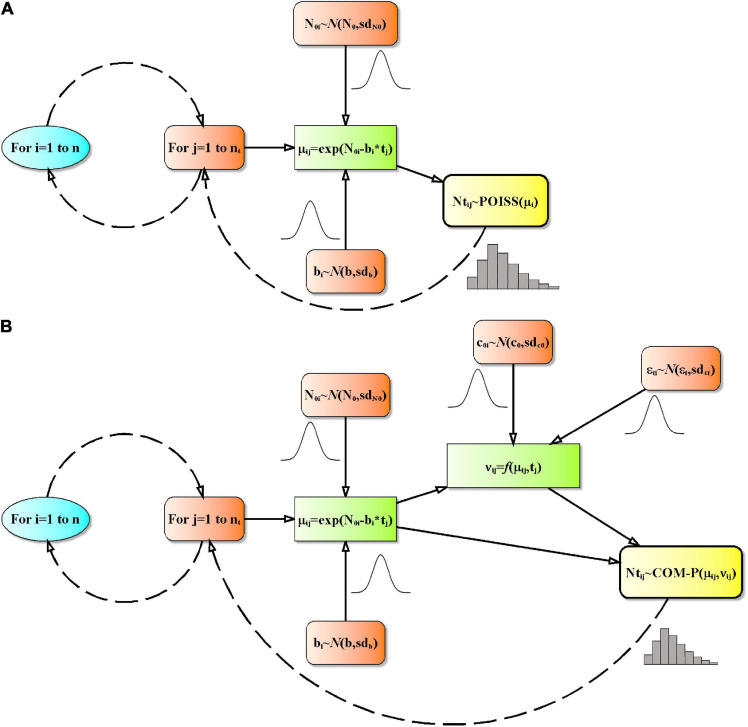
Flowcharts of the computer modeling in the Poisson **(A)** and in the COM-Poisson process (down) **(B)**. Prediction was by Monte Carlo simulation. n: number of simulations; **t_*j*_**, time; **n_*t*_**, number of experimental times; N_0i_: realization of initial count characterized by a normal distribution with mean N_0_ and standard deviation sd_*N0*_; b_*i*_, realization of survival rate characterized by a normal distribution with mean b and standard deviation sd_*b*_; μ**_*ij*_**, centering parameter of the Poisson or COM-Poisson distribution; **N_*tj*_**, realization of **μ_*ij*_**, i.e., the number of survivors; POISS(): Poisson distributed random numbers; ν**_*ij*_**, COM-Poisson dispersion parameter; CMP(): COM-Poisson distributed random numbers; c_0i_, realization of the non-randomness variance parameter characterized by a normal distribution with mean c_0_ and standard deviation sd_*c0*_; **ε_*tij*_**, realization of the additional non-randomness variance parameter characterized by a normal distribution with mean **ε_*t*_** and standard deviation sd**ε_*t*_**.

To predict the *Listeria* inactivation over time a basic Monte Carlo (MC) random sampling with random seed to simulate random numbers from user-provided functions was used (Thomopoulos, 2013). Normal deviates (used to introduce variability in the parameters **N_0_**, **b**, **c_0_**, and **ε_*t*_**) are generated using the Box-Muller Algorithm (Press et al., 1997). Poisson random numbers were generated using the algorithm proposed by Knuth (1997). The COM-Poisson random numbers were generated by the inversion method (Minka et al., 2003). For the expected values ≥200 a normal approximation was used and bounded to integers between 0 and ∞ (Supplementary Table 3). As above reported, the COM-Poisson equations used in random numbers generation were coded, adapting the algorithm proposed by Huntley (2005), in Excel Visual Basic for Application (VBA) (Supplementary Table 3). Inactivation was simulated (Figures 1A,B and Supplementary Table 3) using dispersion of survivors described as randomness in the Poisson process, while randomness, non-randomness and additional non-randomness over time in the COM-Poisson process. For each MC simulation cycle i, a randomly selected inoculum level (**N_0i_**) and a normal random parameter **b_*i*_** was assigned, and at each selected j-th time t_*j*_ the centering parameter **μ_*ij*_** was estimated using Eq. (1) used for inactivation. In the COM-Poisson model **ν_*ij*_** was calculated as a random parameter by applying Eq. (13). Finally, the generation of **N_*tij*_** as random numbers was achieved using Eqs (2) or (3), for the Poisson or COM-Poisson process, respectively.

The number of iterations required to achieve a percentage error of the mean equal to 5% with a 95% level of confidence, was determined by applying MC guidelines (Hahn, 1972; Oberle, 2015). To fulfill these requirements at least 2,300 iterations were required. Monte Carlo simulations, which were recorded by using the mcmon utility in SolverStat (Comuzzi et al., 2003) in Excel, were repeated 5,000 times for each experimental time, assigning 30,000 survival values for each inoculum level assayed. The convergence was assessed by computing confidence intervals on variables of interest (Ballio and Guadagnini, 2004), running in triplicate the MC simulations for different values of the seed of the pseudorandom number generator.

## Results

### Variation in the Initial Cell Number

The initial *Listeria* cell data (N_0_), in consonance with event data, were modelled by both the Poisson and the COM-Poisson distributions (see Supplementary Box 1 for the COM-Poisson regression) and their estimated parameters **a** (**a_*P*_**   and **a_*COM*_**) and **ν** are shown, for each trial, in Table 1. As expected, **a** values were almost identical, at the different initial cell levels in the Poisson and in the COM-Poisson distributions. At the higher counts when N_0_ was around 185/0.2 mL (H samples), the COM dispersion parameter **ν** was < 1, revealing over-dispersion. Accordingly, at these high counts the AIC, which is designed to pick the model that minimizes the information loss, was substantially lower at the higher counts in two trials out of three, while the variance over mean ratios were largely >1 (Table 2). In addition, the *p*-values of the likelihood ratio test (P-LR) were <0.05 in two trials out of three. Likewise, the log-likelihood value (LL), which expresses how many times more likely the data are under one model than the other, was higher at the higher counts in two trials out of three. All these statistics indicated a better fit for the COM-Poisson at the higher counts in two trials out of three assayed. When N_0_ was around 5–20 CFU/0.2 mL (L and M samples), the *p*-values of the likelihood ratio test were larger than 0.05, and LL and AIC were similar in the Poisson and in the COM-Poisson processes (Table 2). In addition, the mean values and variances were similar (the ratios were around 1), showing that for these populations the experimentally obtained counts followed the Poisson distribution.

**TABLE 1:** The Poisson and the COM-Poisson distribution parameters (a_*P*_, Poisson; a_*COM*_, COM-Poisson, **ν** COM-Poisson) of the observed initial bacterial cell counts (LnN_0_: Ln CFU/0.2 mL) in the three trials.

		N_0_ = L			N_0_ = M			N_0_ = H		
		Trial I	Trial II	Trial III	Trial I	Trial II	Trial III	Trial I	Trial II	Trial III
Poisson	a_*p*_	1.37 ± 0.10^*b*^	1.99 ± 0.08	1.49 ± 0.10	2.71 ± 0.08	3.34 ± 0.05	2.83 ± 0.06	4.96 ± 0.03	5.51 ± 0.03	5.11 ± 0.04
COM-Poisson	a_*COM*_	1.39 ± 0.10	1.99 ± 0.07	1.51 ± 0.10	2.69 ± 0.07	3.34 ± 0.05	2.82 ± 0.06	4.96 ± 0.03	5.51 ± 0.03	5.10 ± 0.03
	*v*	1.19 ± 0.53	1.00 ± 0.63	1.12 ± 0.41	0.57 ± 0.19	0.93 ± 0.32	0.85 ± 0.30	0.60 ± 0.19	0.25 ± 0.21	0.28 ± 0.09

*Mean values and standard deviations^*a*^ of *Listeria* initial counts in the three trials were: L = 5.2 ± 1.8 CFU/0.2 mL; M = 20.1 ± 7.2 CFU/0.2 mL; H = 185.1 ± 55.6 CFU/0.2 mL.*

*^*a*^N_0_ standard deviations due to measurement uncertainty were lower than the limit of 0.5 log_10_ CFU/mL (data not shown).*

*^*b*^Standard error.*

**TABLE 2:** Log-likelihood (LL) value, P of likelihood ratio test (P-LR), Akaike information criterion (AIC) and variance-to-mean ratio to determine the better-fitted distribution for the initial observed *L. monocytogenes* counts (LnN_0_: Ln CFU/0.2 mL) in each of the three trials, starting with different initial cells (L, low inoculum; M, medium inoculum; H, high inoculum).

	N_0_ = L			N_0_ = M			N_0_ = H		
	Trial I	Trial II	Trial III	Trial I	Trial II	Trial III	Trial I	Trial II	Trial III
LL^a^ Poisson	–40.34	–48.05	–41.97	–62.77	–59.45	–55.50	–84.61	−**112.70**	−**105.31**
LL COM-Poisson	–40.23	–48.05	–41.91	–60.91	–59.43	–55.37	–83.05	–97.15	–92.19
AIC^b^ Poisson	82.68	98.10	85.93	127.54	120.91	113.00	171.23	227.39	212.62
AIC COM-Poisson	84.45	100.10	87.82	125.81	122.85	114.73	170.11	**198.29**	**188.38**
P-LR^c^	0.64	0.99	0.74	0.05	0.82	0.61	0.08	**0.00**	**0.00**
Variance/Mean^d^	0.95	1.15	1.03	1.76	1.15	1.18	1.75	**4.38**	**3.72**

*^a^If the value of LL is lower, the null hypothesis that the Poisson distribution model is the better model was rejected (in bold).*

*^b^A lower AIC value indicates a better fit of the COM-Poisson distribution model (in bold). Differences greater than 10 indicates that there is essentially no support for the alternative model.*

*^c^If the *P*-value of the likelihood ratio test was smaller than 0.05, the hypothesis that counts followed the Poisson distribution model was rejected (in bold).*

*^d^Equi-dispersion, i.e., the equality between mean and variance of the counts, is a property of the Poisson model distribution. If the ratio is different than 1, the null hypothesis that the Poisson distribution model is the better model was rejected (in bold).*

### Variation in Survivors Over Time

**FIGURE 2:**
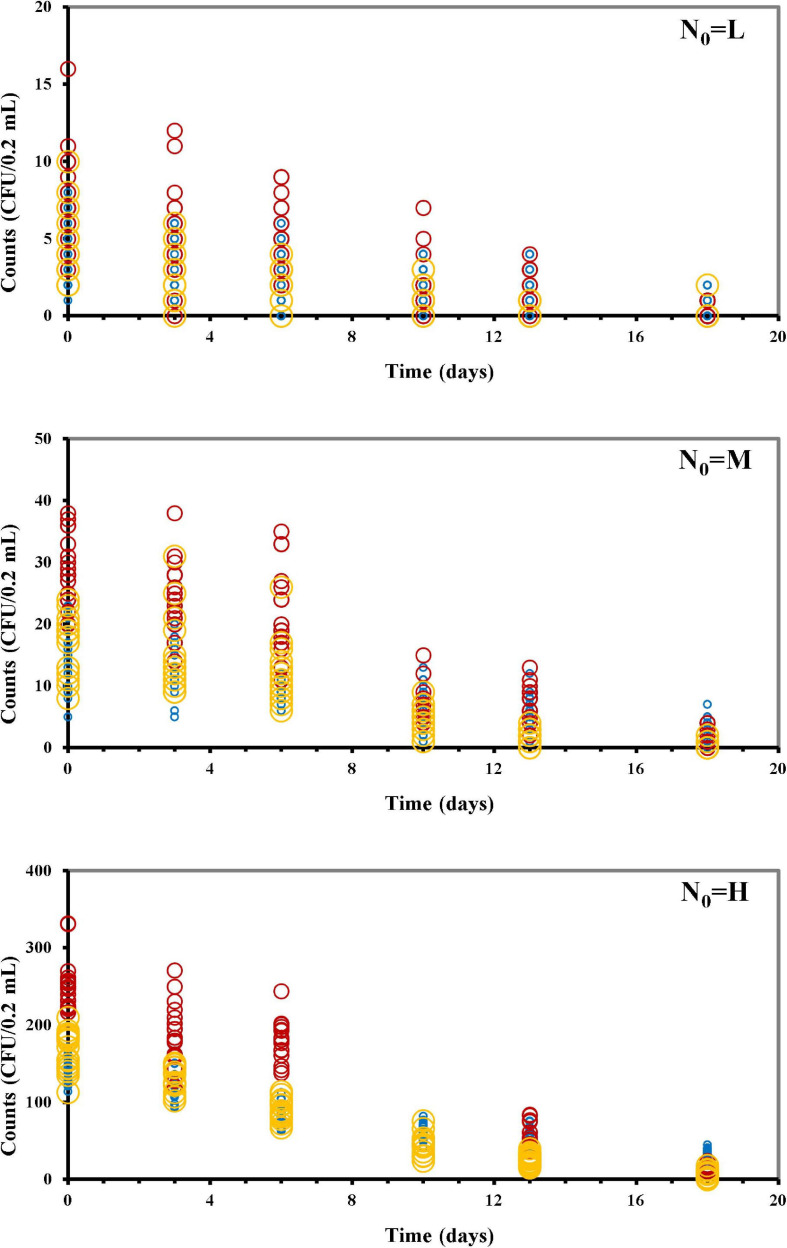
*Listeria monocytogenes* survivors during the osmotic treatment, starting with different initial cells (N_0_: CFU/0.2 mL) (L, low inoculum; M, medium inoculum; H, high inoculum). Trial I, blue circles; trial II, red circles; trial III, yellow circles.

**FIGURE 3:**
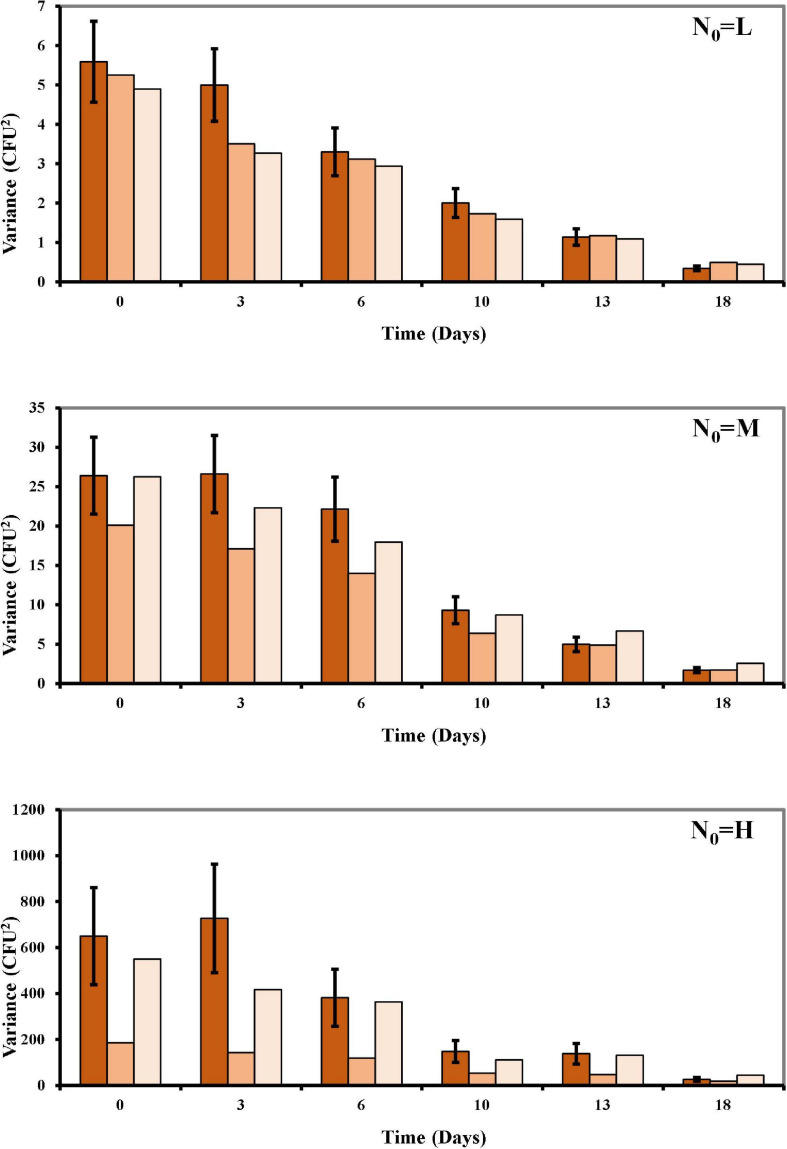
Mean of the variances over time of the observed survival *Listeria* data compared to the Poisson and the COM-Poisson (Supplementary Box 1) distributions fitted to the experimental data points at the different N_0_ (L, low inoculum; M, medium inoculum; H, high inoculum). Observed variances: dark orange columns; Poisson distribution variances: orange columns; COM-Poisson distribution variances: light orange columns; bars are standard errors.

In our study, osmotic inactivation was through an osmotic stress and the resulting *L. monocytogenes* survival data at the different N_0_ in BHI broth at a_*w*_ of 0.913 are shown in Figure 2 and Supplementary Table 1. To describe the variation in the number of surviving cells, both the Poisson and the COM-Poisson regressions (see Supplementary Box 1 for the COM-Poisson regression) were applied to the survival *Listeria* data over time keeping, at this step, **ν** constant for each regression. The trends of the experimental and predicted data variances were then visualized using the mean variance over time at the different N_0_ (Figure 3) to provide visual evidence of the scattering of the mean experimental points and how the COM-Poisson distribution could improve the interpretation of the observed data, at least at the medium and higher counts. This was confirmed by goodness of fit statistics used to determine the better-fitted distribution for *L. monocytogenes* observed survivors in each of the three osmotic inactivation trials, starting with different initial cell levels (Table 3). Starting from the higher count (H) and according to the AIC method the hypothesis that bacterial survivors follow the Poisson distribution was rejected in all the three trials. The LL values and the *p*-values of the LR test (Table 3) confirmed that the COM-Poisson better fit the data in the H samples. The better fit of the COM-Poisson was also observed in two trials out of three of the M samples (II and III). The Poisson model, in which the complexity of computation is reduced, could be appropriate for fitting regression data from the lower count samples (L samples). However, it is not a good choice for data sets where the Poisson assumptions are not met.

**TABLE 3:** Log-likelihood value (LL), P of likelihood ratio test (P-LR) and Akaike information criterion (AIC) to determine the better-fitted distribution for *L. monocytogenes* observed survivors in each of the three osmotic non-thermal inactivation trials, starting with different initial cells (L, low inoculum; M, medium inoculum; H, high inoculum).

	N_0_ = L			N_0_ = M			N_0_ = H		
	Trial I	Trial II	Trial III	Trial I	Trial II	Trial III	Trial I	Trial II	Trial III
LL^a^ Poisson	–207.81	–212.01	–162.29	–296.58	−**322.22**	−**297.45**	−**483.45**	−**701.08**	−**567.28**
LL COM-Poisson	–207.16	–211.59	–162.16	–295.76	–310.47	–288.75	–467.78	–423.07	–435.10
AIC^b^ Poisson	419.61	428.02	328.58	597.15	648.44	598.89	970.90	1406.16	1138.55
AIC COM-Poisson	420.33	429.19	330.33	597.51	**626.94**	**583.50**	**941.56**	**852.15**	**876.19**
P-LR^c^	0.26	0.36	0.62	0.20	**0.00**	**0.00**	**0.00**	**0.00**	**0.00**

*At the initial steps of the modeling, the COM-Poisson (Supplementary Box 1) dispersion parameter **ν** was assumed as independent of the bacterial counts.*

*^a^If the value of LL is lower, the null hypothesis that the Poisson distribution model is the better model was rejected (in bold).*

*^b^A lower AIC value indicates a better fit of the COM-Poisson distribution model (in bold). Differences greater than 10 indicates that there is essentially no support for the alternative model.*

*^c^If the *P*-value of the likelihood ratio test was smaller than 0.05, the hypothesis that counts followed the Poisson distribution model was rejected (in bold).*

The Poisson and the COM-Poisson inactivation rate parameters (**b**) and their associated statistics were calculated and reported in Table 4, along with the COM-Poisson dispersion parameter **ν** which is assumed, at this step, as independent of the microbial counts. The inactivation parameters (mean values from the 9 trials: −0.120 ± 0.035 day^–1^ and −0.125 ± 0.035 day^–1^ for the Poisson and COM-Poisson, respectively) were not affected (*p* > 0.05) by the initial cell numbers, but by the trials (*p* < 0.05), indicating the relevance of variability in population dynamics in the case of microbial inactivation. For this reason, in the stochastic inactivation model (see section “Stochastic Inactivation Modeling and Comparison With Random Numbers Generated in a Computer Simulation”) normal distributions with their parameters (mean and standard deviations as reported above) were used to describe the variation of the inactivation rates. For each trial the degree of dispersion of the inactivation parameters, expressed as standard error, decreased as the initial population increased. It therefore appeared that the smaller the population, the more spread out the linear rate of inactivation was, due to stochastic variation. On the other hand, the cells that survived the osmotic treatment starting from the highest values and the medium initial counts (H and M samples) showed most of the values of the COM-Poisson dispersion parameter **ν** < 1, revealing over-dispersion. The **ν** decline was similar (data not shown) when testing much higher initial cell levels, which, however, required serial dilutions to perform the experiments (Supplementary Table 1, HH data).

**TABLE 4:** Osmotic inactivation rate (b) (day^–1^) parameters of *L. monocytogenes* at different initial cells (L, low inoculum; M, medium inoculum; H, high inoculum) and the COM-Poisson (Supplementary Box 1) dispersion parameter **ν** (assumed, at this step, as independent of the bacterial counts) obtained by the Poisson and COM-Poisson regressions.

		N_0_ = L			N_0_ = M			N_0_ = H		
		Trial I	Trial II	Trial III	Trial I	Trial II	Trial III	Trial I	Trial II	Trial III
Poisson	b	−0.0811 ± 0.0120^a^	−0.1179 ± 0.0103	−0.1743 ± 0.0165	−0.0860 ± 0.0067	−0.1186 ± 0.0060	−0.1589 ± 0.0090	−0.0789 ± 0.0024	−0.1199 ± 0.0058	−0.1487 ± 0.0048
COM-Poisson	b	−0.0871 ± 0.0148	−0.1218 ± 0.0130	−0.1675 ± 0.0207	−0.0872 ± 0.0066	−0.1235 ± 0.0065	−0.1728 ± 0.0105	−0.0794 ± 0.0021	−0.1259 ± 0.0050	−0.1558 ± 0.0068
	ν	0.8093 ± 0.1196	0.8574 ± 0.1708	1.1089 ± 0.2539	0.8403 ± 0.1343	0.5263 ± 0.0879	0.5548 ± 0.1352	0.5228 ± 0.0729	0.0999 ± 0.0163	0.1830 ± 0.0246

*^a^Standard error.*

The aggregation assay, which is based on bacterial sedimentation, was performed by measuring the optical absorbance of culture supernatant. With this assay, the aggregative ability of *L. monocytogenes* (Travier et al., 2013; Eshwar et al., 2017) was substantiated in the presence of salt and over time and enhanced (*p* < 0.05) in the osmotic medium (Supplementary Figure 1A). In the latter, the aggregative ability increased proportionally to the cell density (Supplementary Figure 1B). Although cell densities required for measuring auto-aggregation through the culture absorbance shift were higher than those used in our modeling experiments, these results supported the idea that aggregation during osmotic stress occurred in a concentration-dependent manner. Since aggregation can result in a clustered distribution with variance greater than its mean (over-dispersion), it is conceivable to hypothesize a role of cell-density mediated aggregation in over-dispersion, which was more relevant at the highest counts (H samples).

### Stochastic Modeling of Dispersion

**FIGURE 4:**
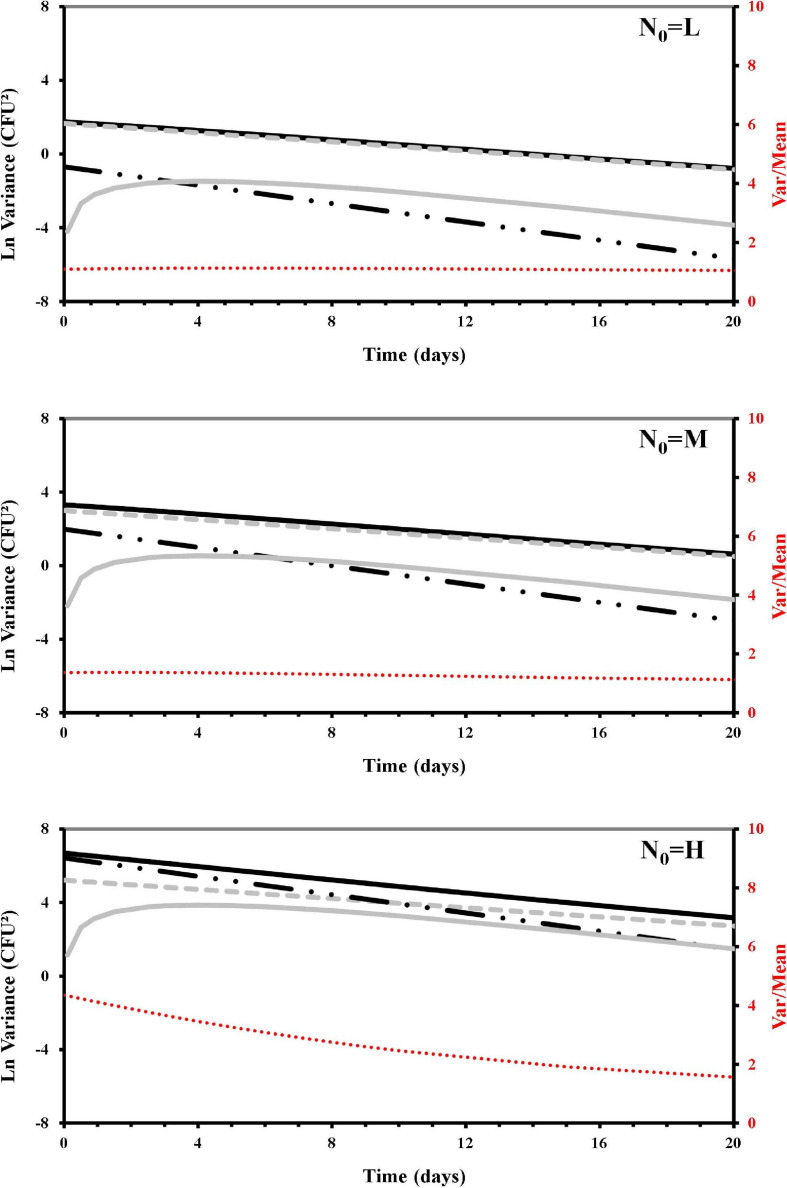
Simulated contributors to variance of *Listeria monocytogenes* survivors during the osmotic treatment, starting with different initial cells (L, low cell density; M, medium cell density; H, high cell density). Total variance (*Var*[N_*t*_]): black solid line; Poisson variance *E*[N_*t*_]: dashed gray line; non-randomness c_0_*E*[N_*t*_]^2^: dashed black line; additional contribution (due to the osmotic treatment) to non-randomness ε_*t*_t *E*[N_*t*_]^2^/*E*[N_0_]^1/2^: gray solid line; variance over mean (secondary axis): dashed red line.

To quantify the variance contributors producing over-dispersion, we developed a model for **ν** [see section “Materials and Methods”: Eq. (13)] that through the **c_0_** and **ε_*t*_** parameters (Table 5) estimated the total variance and distinguished its contributors in terms of randomness, non-randomness, and an additional non-randomness arising during the osmotic treatment. This modeling approach allowed us to integrate and distinguish these different components of variation into simulations that are shown in Figure 4. The contribution of randomness to the total variance was confirmed to be dominant in the lower count (L samples) survivors of the osmotic inactivation procedure, where the non-randomness contribution to the variance, even that due to the osmotic treatment, was almost irrelevant. In the medium count survivors (M samples) the randomness contribution to variance was always dominant, but non-randomness, even that due to the osmotic treatment, was larger than in the L survivors. For both L and M samples the variance-to-mean ratios were around one. At the higher counts (H samples) the non-randomness increased the total variance above the Poisson distribution, making the osmotic non-randomness contribution more relevant. On the other hand, during the osmotic treatment, along with the decreasing number of survivors, the randomness tended to overtake the non-randomness contribution with the duration of the treatment. In these H samples the variance-to-mean ratio, initially predicted about four times larger than the mean, tended to be closer to the mean.

**TABLE 5:** Estimation and related regression statistics of **ν** model (Eq. 13) parameters.

Parameter	Value	Std. Err.	Goodness of fit tests		Log-likelihood	
**c${}_{0}^{\text{a}}$** (CFU^–1^)	0.0181^b^	0.0039	Pseudo *R*^2^	0.9737	Maximum log-likelihood	−1948.11
**ε_*t*_** (CFU^–1/2^day^–1^)	0.0128^b^	0.0024	Adjusted Pseudo *R*^2^	0.9737	Intercept-Only Model	−36462.88
			RMSE (Root Mean Squared Error)	13.73	Fitted Model	−2854.80
			SEE (Standard Error of Estimates)	13.74		

*^a^C_0_ < 2e for e ≥ 0.01 (Supplementary Box 2).*

*^b^Statistically significant (*p* < 0.05).*

From the goodness of fit results (Table 6), it was evident that the Poisson and the COM-Poisson processes were quite distinct in terms of their ability to capture variance. The COM-Poisson overall performance, which was better than the Poisson, could be attributable to its ability to deal with over-dispersion exhibited by the higher counts.

**TABLE 6:** Log-likelihood value (LL) and Akaike information criterion (AIC) values to determine the better-fitted distribution between the Poisson and the COM-Poisson (eq. 13) processes for *L. monocytogenes* observed survivors starting with different initial cells (L, low inoculum; M, medium inoculum; H, high inoculum).

	Poisson			COM-Poisson (Eq. 13)		
	LL^a^	p^b^	AIC^c^	LL	*p*	AIC
**N_0_ = L^*d*^**	−582.11	6	1176.22	**577.96**	8	1171.93
**N_0_ = M^*d*^**	−916.24	6	1844.49	**887.24**	8	**1790.48**
**N_0_ = H^*d*^**	1751.81	6	3515.61	**1389.60**	8	**2795.20**
**ALL^*e*^**	3250.16	18	6536.32	**2854.80**	20	**5749.61**

*^a^If the value of LL is lower, the null hypothesis that the Poisson distribution model is the better model was rejected (in bold).*

*^b^Parameter numbers.*

*^c^A lower AIC value indicates a better fit of the COM-Poisson distribution model (in bold). Differences greater than 10 indicates that there is essentially no support for the alternative model.*

*^d^Value from the three trials.*

*^e^Value from the nine trials.*

Further diagnostic analysis of the COM-Poisson and Poisson processes was done applying the regression residual deviance. The null deviance, referred to the null model, which shows how well the response variable is predicted by a model including only the intercept, amounted to 69020.53 on 992 degrees of freedom (DF). Adding the variance components that are comprised in the Poisson and COM-Poisson frameworks the residual deviance, which represents the quantity of variation unexplained by the model, significantly decreased to 2604.10 (on 975 DF) and 1813.39 (on 973 DF), respectively. Hence, taking into account variation in the number of survivors, which were assigned to a combination of biological variability and uncertainty, the explanatory power of the processes increased, with the COM-Poisson providing a better description of the variance. It is worth noticing that the quantity of variation explained by the COM-Poisson process also encompassed over-dispersion.

### Stochastic Inactivation Modeling and Comparison With Random Numbers Generated in a Computer Simulation

Monte Carlo, which is a common method to approximate the distribution of a model output, has been successfully used to describe variability and uncertainty in survival numbers (Akkermans et al., 2018; Abe et al., 2019; Garre et al., 2019). MC simulations were then used to model the variation in survivors at various initial cell counts in both the Poisson and the COM-Poisson frameworks (MC-Poisson and MC-COM), with the latter including the dispersion parameter **ν** (Table 7). Most of the observed values were within the MC estimated-ranges of the counts predicted in both processes. It is worth noticing that in the MC-COM the variations estimated at the higher counts were larger than those estimated in the MC-Poisson framework, which assumes only randomness. Since the dispersion parameter was successfully predicted by MC simulations, it could be inferred that other contributors to variance, i.e., non-randomness and an additional non-randomness emerging during the osmotic treatment, could have contributed to the observed differences in variations (Table 7). Therefore, the additional variance components in the observed values could be substantiated by the Monte Carlo within the COM-Poisson framework, which was able to describe the randomness and non-randomness bacterial behavior.

**TABLE 7:** Observed and Monte Carlo simulation results for osmotic inactivation of *L. monocytogenes* starting with different initial cells (L: low inoculum, M: medium inoculum, H: high inoculum).

Time (Days)	Observed survivors^a^	COM-Poisson simulation^b^	Poisson simulation^c^	Observed v^d^	Simulated v^e^
**N_0_ = L**					
0	5.0±2.2f	5.0±2.4	5.0±2.3	0.97±0.09	0.92±0.03
3	3.0±2.4	3.0±2.1	3.0±2.1	0.74±0.11	0.89±0.03
6	3.0±1.9	2.0±2.0	2.0±1.9	1.06±0.50	0.89±0.04
10	1.0±2.0	1.0±1.9	1.0±1.9	1.02±0.37	0.90±0.04
13	1.0±1.8	1.0±1.9	1.0±1.9	1.18±0.42	0.92±0.05
18	0.0±1.8	0.0±1.9	0.0±1.9	1.34±0.77	0.94±0.05
**N_0_ = M**					
0	19.0±4.8	20.0±5.3	20.0±4.5	0.76±0.17	0.74±0.08
3	15.0±5.5	14.0±5.2	14.0±4.7	0.70±0.26	0.73±0.07
6	11.0±5.1	9.0±5.6	9.0±5.3	1.62±1.91	0.75±0.07
10	6.0±5.6	5.0±6.3	5.0±6.1	0.78±0.29	0.79±0.08
13	4.0±5.5	4.0±6.5	4.0±6.4	1.11±0.32	0.83±0.08
18	1.0±6.2	2.0±6.8	2.0±6.8	0.93±0.17	0.88±0.09
**N_0_ = H**					
0	171.5±22.9	182.0±30.0	184.5±13.6	0.36±0.19	0.23±0.08
3	133.0±32.9	124.0±32.2	127.0±24.9	0.34±0.21	0.28±0.08
6	97.0±23.0	85.0±39.5	86.0±36.7	0.40±0.16	0.33±0.09
10	54.0±23.7	51.0±46.3	52.0±45.5	0.42±0.27	0.41±0.11
13	46.5±46.2	35.0±49.4	35.0±48.8	0.43±0.17	0.48±0.13
18	17.5±53.1	19.0±52.4	19.0±52.1	0.73±0.50	0.60±0.15

*^a^Median values.*

*^b^The COM-Poisson framework was used to predict the *Listeria* inactivation using Monte Carlo simulation.*

*^c^The Poisson framework was used to predict the *Listeria* inactivation using Monte Carlo simulation.*

*^d^Observed COM-Poisson dispersion parameter.*

*^e^Simulated COM-Poisson parameter.*

## Discussion

Dispersion of pathogenic microorganisms in food has a strong impact on public health (Jongenburger et al., 2012a). It is therefore of importance to provide a framework that can be used to represent and distinguish the randomness and the non-randomness components of variation. The COM-Poisson process, which refers to COM-Poisson distribution, can be a good candidate. In fact, the two-parameter COM-Poisson distribution, which has the Poisson distribution as a special case, can deal with both the over-dispersed (**ν** < 1) and under-dispersed (**ν** > 1) count data (Sellers and Shmueli, 2010; Francis et al., 2012), whereas the Poisson distribution has only one parameter, which represents both the expectations and variance of the count random variable. In addition, unlike the Poisson model where the conditional mean is central to interpretation, the COM-Poisson distribution, taking into account the complete conditional fitted distribution, uses a more general function of the response distribution (Sellers and Shmueli, 2010).

According to our results, and as reported by others (Koyama et al., 2016, 2019), initial small cell numbers followed the Poisson distribution, indicating that they exhibited naturally occurring randomness. On the other hand, larger amounts of cells mostly followed the COM-Poisson distribution, revealing over-dispersion. It is well known that only for low-density populations the cells can be randomly spread, whereas the frequent presence of clumps and aggregates in larger populations could result in the detection of over-dispersed data. However, in this context, it can be more accurate to model the over-dispersed microbial data under the theoretical interpretation of independent events and not to follow a true contagious process of non-independent events (Gonzales-Barron and Butler, 2011). Dispersion was then confirmed to be dependent on the initial number of cells, i.e., as the number of initial cells increased, the randomness contribution to the variance decreased, while over-dispersion increased.

Similarly to what was observed by others (Aspridou and Koutsoumanis, 2015), in the experiments conducted to generate the osmotic inactivation rates, number of cells, had no significant effect on the inactivation rates, possibly due to the lack of a cooperative behavior (García and Cabo, 2018). On the other hand, a trial effect was observed, suggesting that such effect could depend on each baseline population history, which determines both regulatory and mutational responses to new environments (Ryall et al., 2012). The biological individuality, which refers also to cell to cell variations from a given species, is therefore of great importance in the case of microbial inactivation (Aspridou and Koutsoumanis, 2015, 2020). Unlike what was observed for the rate of inactivation, the dispersion parameter **ν** did not respond to determinants other than the cell levels, reinforcing the view of the importance of the number of cells as effectors of over-dispersion.

Following inactivation, the stochastic variation dominated in the smaller populations. However, even randomly dispersed populations may allow for the survival of over-dispersed cell populations. Thus, the results on the survival *Listeria* cells ultimately justified the use of the COM-Poisson over the Poisson distribution in its ability to fit differently dispersed count data and sustained the idea that when an osmotic treatment is applied it allows for the survival of over-dispersed cell populations. This additional contribution to the total variance in terms of non-randomness, noticed in the populations following the osmotic stress, is consistent with the hypothesis that over-dispersion could be due to aggregation. *L. monocytogenes* aggregation is mediated by key virulence determinants and can represent a strategy for surviving in inimical environments as those at high NaCl concentration (Jensen et al., 2007; Travier et al., 2013; Eshwar et al., 2017). Hence, the non-randomness, attributable to bacterial abundance, could also arise following an osmotic stress that can contribute to cell aggregation (Schmid et al., 2009).

Quantification of the variance contributors of over-dispersion through the **ν** (dispersion) model allowed us to integrate the different components of variation into the COM-Poisson inactivation process. This latter process enabled the description of the survival of different-sized populations by introducing the COM-Poisson distribution for survivors along with the modelled COM-Poisson dispersion parameter (**ν**). Although a number of stochastic models describing the population randomness under inactivation have been developed (Aguirre et al., 2009; Koyama et al., 2017; Abe et al., 2019; Hiura et al., 2020), less attention has been paid to modeling other variance components, such as the non-randomness (Reinders et al., 2003, 2004; Gonzales-Barron and Butler, 2011). Thus, we proposed a statistical modeling approach, suitable for count data, for accurately estimating the variation in microbial response to an osmotic inactivation and to capture the randomness and non-randomness contributions to the total variance that can have practical implications when dealing with intervention strategies capable of controlling pathogens. The suitability of the approach was demonstrated by its flexibility in handling different dispersion types addressing the variance contributors in different-sized populations. The variation in bacterial numbers, as defined in this study in the context of osmotic stress, and the notions of the random and non-random occurrence of surviving bacteria, could be applied to other hurdles or processes, i.e., thermal processing, used to inactivate bacteria for managing food safety in more realistic conditions.

## Data Availability Statement

The original contributions presented in the study are included in the article/Supplementary Material, further inquiries can be directed to the corresponding author/s.

## Author Contributions

MS and PP: conception and design of the study. MD: acquisition, analyses, and interpretation of data. MS: drafting of manuscript. MS, MD, and PP: critical revision and final approval of the version to be submitted. All authors contributed to the article and approved the submitted version.

## Conflict of Interest

The authors declare that the research was conducted in the absence of any commercial or financial relationships that could be construed as a potential conflict of interest.

## Supplementary Material

Supplementary Figure 1 Quantification of the autoaggregation ability (%) of *L. monocytogenes* in PBS and in osmotic PBS: **(A)** effect of salt; **(B)** effect of initial optical density.

Supplementary Table 1 Excel workbook containing observed count data.

Supplementary Table 2 Excel workbook containing Poisson and COM-Poisson regressions as examples.

Supplementary Table 3 Excel workbook containing Poisson and COM-Poisson random numbers routines.

Supplementary Box 1 Conway-Maxwell-Poisson Regression.

Supplementary Box 2 Equation (8, 9) in the text.

Supplementary Box 3 Empirical approach for selecting the additional non-randomness model component.

## Abbreviations

AIC: Akaike information criterion
a_*w*_: water activity
CFU: colony forming units
COM-Poisson: Conway-Maxwell Poisson
DF: degrees of freedom
H: high inoculum
L: low inoculum
LL: log- likelihood
LR: likelihood ratio
M: medium inoculum
MC: Monte Carlo
NTI: non-thermal inactivation
OD: optical density
P-LR: *p*-values of the likelihood ratio
QMRA: quantitative microbial risk assessment.
