# Supplementary figures and images for: The COM-Poisson Process for Stochastic Modeling of Osmotic Inactivation Dynamics of Listeria monocytogenes
Source: Front Microbiol. 2021 Jul 9;12:681468. doi: 10.3389/fmicb.2021.681468 (PMC8300431; doi:10.3389/fmicb.2021.681468)

Supplementary Material

# Figure S1

| 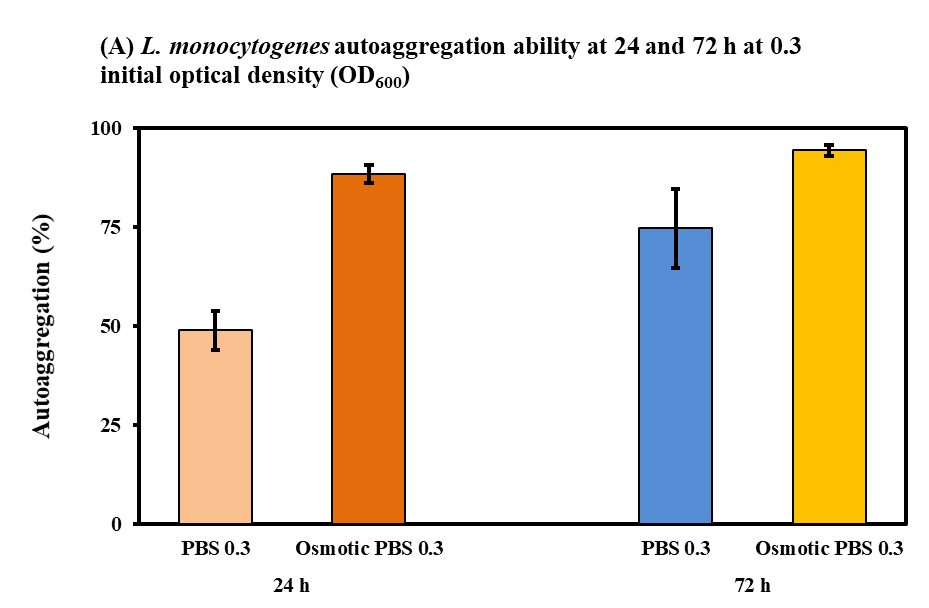 |
| --- |
| 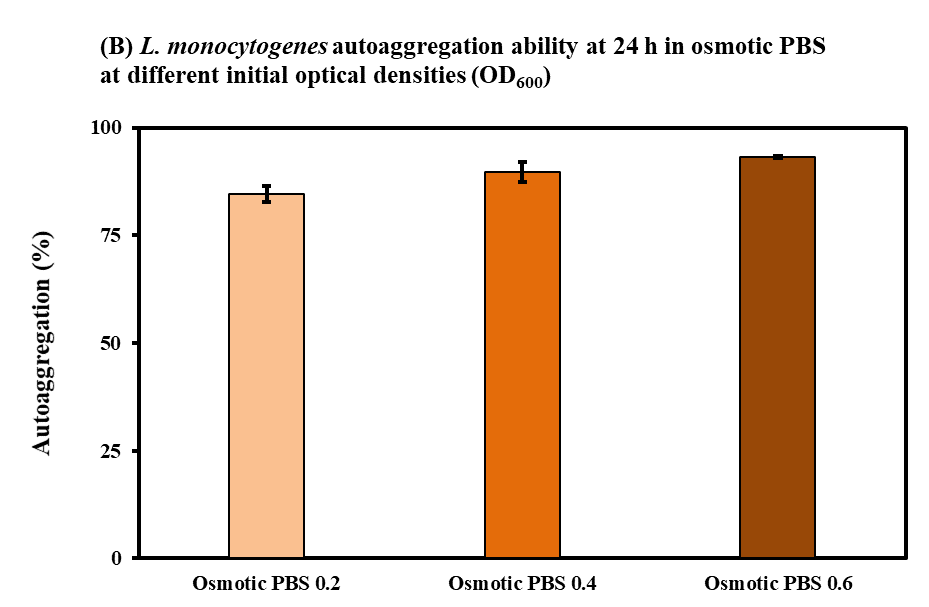 |

Supplement: Supplementary Figure 1 — Quantification of the autoaggregation ability (%) of L. monocytogenes in PBS and in osmotic PBS: (A) effect of salt; (B) effect of initial optical density. [file Data_Sheet_1.docx]
