# Supplementary material for: The COM-Poisson Process for Stochastic Modeling of Osmotic Inactivation Dynamics of Listeria monocytogenes
Source: Front Microbiol. 2021 Jul 9;12:681468. doi: 10.3389/fmicb.2021.681468 (PMC8300431; doi:10.3389/fmicb.2021.681468)
Supplement: Supplementary Box 1 — Conway-Maxwell-Poisson Regression. [file Data_Sheet_2.docx]

Supplementary Material

# Box S1: Conway-Maxwell-Poisson Regression

A two-parameter generalization of the Poisson distribution was introduced by Conway and Maxwell (1962). This distribution, now known as the COM-Poisson distribution, can model data that are either under- or over-dispersed relative to the Poisson distribution (Sellers and Shmueli, 2010; Shmueli et al., 2005). It is characterized by two parameters: **λ** is the location parameter and **ν** is the dispersion parameter that outlines deviations from Poisson distribution. Value of **ν** corresponds to under-dispersion if **ν** > 1 and over-dispersion if **ν** < 1; **ν** = 1 yields the Poisson distribution.

In the original formulation, the probability mass function for the COM-Poisson is given by the equation (S1):

| $\Pr(\text{Y}=\text{y}\vert\text{λ}\text{,}\text{ν})=\frac{\text{λ}^{\text{y}}}{\left( \text{y!} \right)^{\text{ν}}\text{Z}\left( \text{λ}\text{,}\text{ν} \right)},\text{ y}=\text{0,1,2,...}$ $Z\left( \text{λ}\text{,}\text{ν} \right)=\sum_{j=0}^{\infty} \frac{\text{λ}^{\text{j}}}{\left( \text{j!} \right)^{\text{ν}}}$ | (S1) |
| --- | --- |

Where Pr(Y = y| λ,ν) is the conditional probability mass function of the event Y = y, given the parameters λ and ν; *Z*(λ,ν) is a normalizing factor.

Mean and variance are estimated by (S2):

| $E[\text{Y}]\approx\text{λ}^{1/\text{ν}}+\frac{1}{\text{2}\text{ν}}-\frac{1}{2},\text{ }VAR[\text{Y}]\approx\frac{1}{\text{ν}}\text{λ}^{1/\text{ν}}$ | (S2) |
| --- | --- |

where *E*[Y] is the expectation (or mean) for the event Y and *VAR*[Y] is its variance.

A limitation of the COM-Poisson model is that neither **λ** nor **ν** represent a clear centering parameter. To overcome this drawback Guikema & Goffelt (2008) proposed a parametrization of the original COM-Poisson model, using the relation (S3):

| $\text{μ}=\text{λ}^{1/\text{ν}}$ | (S3) |
| --- | --- |

With this parametrization, **µ** is a reasonable approximation of the mean, while **ν** keeps its role as a shape parameter.

Using this parametrization, a maximum-likelihood estimation method was used for fitting the COM-Poisson under a generalized linear model (GLM) framework, as follows (S4):

| $E[\text{Y }\vert\text{ X}]=\text{μ}+\frac{1}{\text{2}\text{ν}}-\frac{1}{2}=\text{EXP}\left( \text{β}_{0}+\sum_{\text{i=1}}^{\text{p}} \text{β}_{\text{i}}\text{x}_{\text{i}} \right)$ $\text{ν}=f\left( \text{X} \right)$ | (S4) |
| --- | --- |

Where ***E*[Y|X]** is the expectation of the count random variable Y, given the set X of independent variables x_i_; **β_0_** and **β_i_** are regression parameters, **μ** is the centering parameter and **ν** is the shape parameter, which can be a constant or a function.

With this formulation, the shape parameter **ν** can be open to a second link function to allow the amount of over-dispersion or under-dispersion to vary across measurements.

For large values of variable Y or for ν close to 1, equation (S4) may be reduced to equation (S5)

| $E[\text{Y }\vert\text{ X}]\approx\text{μ}=\text{EXP}\left( \text{β}_{\text{0}}+\sum_{\text{i=1}}^{\text{p}} \text{β}_{\text{i}}\text{x}_{\text{i}} \right)$ $\text{ν}=f\left( \text{X} \right)$ | (S5) |
| --- | --- |

For N observations, the log-likelihood for the GLM model can be written as (S6):

| $\text{L}\left( \text{Y}\vert\text{X},\text{β},\text{ν} \right)=\sum_{\text{i=1}}^{\text{N}} \text{y}_{\text{i}}\ln\left( \text{λ}_{\text{i}} \right)-\sum_{\text{i=1}}^{\text{N}} \text{ν}\text{ ln}\left( \text{y}_{\text{i}}! \right)-\sum_{\text{i=1}}^{\text{N}} \text{ln}\left( Z\left( \text{λ}_{\text{i}}\text{,}\text{ν} \right) \right)$ | (S6) |
| --- | --- |

Where L(Y|X, β,ν) is the log-likelihood of the set Y, given X, β, ν; L(Y|X, β, ν) is the objective-function that should be maximized during GLM regression; y_i_ is the observed value, λ_i_ is the location parameter calculated from function (S3) and ν is the shape parameter.

Note that the likelihood function of the COM-Poisson regression model, differently from the Poisson model, includes the **ν** parameter. It follows that the log-likelihood functions could be quite different even when the β parameters are similar.

# References

Conway, R.W., & Maxwell, W.L., (1962). A queueing model with state dependent service rate. Journal of Industrial Engineering, 12, 132-136.

Guikema, S.D., & Goffelt, J.P. (2008). A flexible count data regression model for risk analysis. Risk Analysis, 28, 213-223.

Sellers, K.F., & Shmueli, G. (2010). A flexible regression model for count data. Annals of Applied Statistics, 4, 943-961. https://doi.org/10.1214/09-AOAS306.

Shmueli, G., Minka, T., Kadane, J.B., Borle, S., & Boatwright, P.B. (2005). A useful distribution for fitting discrete data: revival of the Conway–Maxwell–Poisson distribution. Journal of the Royal Statistical Society, Series C, 54, 127-42.

# Box S2: text Equations (8) and (9)

Equation (8), which is a second-degree polynomial equation, can be solved for ***E***[**N_t_]** and **v** (wxMaxima, Version 17.10.1, <http://wxmaxima.sourceforge.net/>), but solutions (S7 and S8) are not very comfortable:

| $\left[ \begin{matrix} \text{ν}_{1}=\frac{2E\left[ \text{N}_{\text{t}} \right]+1-\sqrt{4E\left[ \text{N}_{\text{t}} \right]^{2}-8\text{c}_{\text{0}}E\left[ \text{N}_{\text{t}} \right]^{2}-4E\left[ \text{N}_{\text{t}} \right]+1}}{4\text{c}_{\text{0}}E\left[ \text{N}_{\text{t}} \right]^{2}+4E\left[ \text{N}_{\text{t}} \right]}, \\ \text{ν}_{2}=\frac{2E\left[ \text{N}_{\text{t}} \right]+1+\sqrt{4E\left[ \text{N}_{\text{t}} \right]^{2}-8\text{c}_{\text{0}}E\left[ \text{N}_{\text{t}} \right]^{2}-4E\left[ \text{N}_{\text{t}} \right]+1}}{4\text{c}_{\text{0}}E\left[ \text{N}_{\text{t}} \right]^{2}+4E\left[ \text{N}_{\text{t}} \right]} \end{matrix} \right]$ | (S7) |
| --- | --- |

and

| $\left[ \begin{matrix} E\left[ \text{N}_{\text{t}} \right]_{1}=\frac{1-\text{ν}+\sqrt{\text{ν}^{2}+2\text{ν}\left( \text{c}_{\text{0}}-1 \right)-2\text{c}_{\text{0}}+1}}{2c_{0}\text{ν}}, \\ E\left[ \text{N}_{\text{t}} \right]_{2}=\frac{1-\text{ν}-\sqrt{\text{ν}^{2}+2\text{ν}\left( \text{c}_{\text{0}}-1 \right)-2\text{c}_{0}+1}}{2\text{c}_{0}\text{ν}} \end{matrix} \right]$ | (S8) |
| --- | --- |

However, under certain circumstances, Equation (8) can be simplified. When **ν** approaches to 1, the term **1/(2 ν) -1/2** become null and Equation (8) is reduced to Equation (9). When the term **1/(2 ν) -1/2** corresponds to the maximum acceptable absolute error **e*E* [N_t_],** we can write (S9):

| $\text{e}E\left[ \text{N}_{\text{t}} \right]=\frac{1}{\text{2}\text{ν}}-\frac{1}{2}$ | (S9) |
| --- | --- |

Where **e** is the maximum relative acceptable error.

Then:

| $\text{2e}E\left[ \text{N}_{\text{t}} \right]=\frac{1}{\text{ν}}-1$ | (S10) |
| --- | --- |

| $\text{1+2e}E\left[ \text{N}_{\text{t}} \right]=\frac{1}{\text{ν}}$ | (S11) |
| --- | --- |
| $\frac{1}{\left( \text{1+2e}E\left[ \text{N}_{\text{t}} \right] \right)}=\text{ν}$ | (S12) |

replacing **ν** in Equation (9) we have (S13):

| $E\left[ \text{N}_{\text{t}} \right]=\frac{1}{\left( \text{1+2e}E\left[ \text{N}_{\text{t}} \right] \right)}\left( E\left[ \text{N}_{\text{t}} \right]+\text{c}_{0}E\left[ \text{N}_{\text{t}} \right]^{2} \right)$ | (S13) |
| --- | --- |

| $E\left[ \text{N}_{\text{t}} \right]=E\left[ \text{N}_{\text{t}} \right]\frac{\left( 1+\text{c}_{\text{0}}E\left[ \text{N}_{\text{t}} \right] \right)}{\left( \text{1+2e}E\left[ \text{N}_{\text{t}} \right] \right)}$ | (S14) |
| --- | --- |

| $1=\frac{\left( 1+\text{c}_{0}E\left[ \text{N}_{\text{t}} \right] \right)}{\left( \text{1+2e}E\left[ \text{N}_{\text{t}} \right] \right)}$ | (S15) |
| --- | --- |

From (S15) it is evident that **c_0_ = 2 e**. Then, when **c_0_ ≤ 2 e**, Equation (8) can be simplified to Equation (9), as in the text.

# Box S3: Empirical approach for selecting the additional non-randomness model component

In response to the osmotic treatment a transitory additional variation, not explained by the equation 7 and possibly attributable to aggregation, occurred over time. This additional variation was higher at the beginning of the process and then decreased along with the decreasing of the number of survivors. Therefore, the appearance of this variation and its behaviour during treatment required the introduction of an additional model component, defined as “additional non-randomness” variation. To describe this component, a number of empirical equations were developed (Table 1-Box S3) and the Akaike information criterion (AIC) was used to select the best fit model under parsimony. AIC was calculated using the number of the fitted parameters and the number of components included in the additional non-randomness variation. A single model, which gave the best fit to the data, was chosen.

**Table 1-Box S3. Different models and their AIC**

| **c_0_** | **ε_t_** | **t^** | ***E*[N_t_]^** | **1/*E*[N_0_]^** | **LL** | **n** | **p** | **AIC** |  |
| --- | --- | --- | --- | --- | --- | --- | --- | --- | --- |
| 0.0181 | 0.0128 | 1 | 2 | 0.5 | -2854.8 | 993 | 5 | 5719.6 | * |
| 0.0315 | 0.0181 | 1 | 1 | -1 | -2833.7 | 993 | 5 | 5677.4 | ** |
| 0.0234 | 0.0290 | 1 | 1.5 | -- | -2845.6 | 993 | 4 | 5699.2 | ** |
| 0.0346 | 0.0487 | 1 | 1 | -- | -2848.4 | 993 | 4 | 5704.7 | ** |
| 0.0183 | 0.0060 | 1 | 2 | -- | -2855.4 | 993 | 4 | 5718.7 | ** |
| 0.0190 | 0.0255 | 1 | 2 | 1 | -2855.2 | 993 | 5 | 5720.4 | ** |
| -- | -- | -- | 1 | 0.5 | -- | 993 | 4 | -- | *** |
| 0.0269 | 0.0648 | 1 | 2 | 2 | -2860.6 | 993 | 5 | 5731.2 | **** |
| 0.0339 | 0.0044 | 2 | 2 | 2 | -2864.0 | 993 | 5 | 5737.9 | **** |
| 0.0405 | 0.0616 | 1 | 1 | 1 | -2865.0 | 993 | 5 | 5739.9 | **** |
| 0.0455^$^ | -- | -- | -- | -- | -2870.7 | 993 | 1 | 5743.4 | **** |

*selected model (equation 12)

**models excluded for variance to mean ratio unrealistic prediction

***models excluded for lack of convergence

**** models excluded for AIC performance

n: numbers of data

p: numbers of parameters and components
